# How many psychiatric beds are needed—and for what?

**DOI:** 10.1007/s00115-025-01875-x

**Published:** 2025-08-25

**Authors:** Stefan Priebe

**Affiliations:** Centre for Psychosocial Medicine, University Medical Center Hamburg-Eppendorf, Hamburg, Germany

**Keywords:** Mental health, Evaluation, Psychiatric illness, Hospital bed capacity, Length of stay, Psychische Gesundheit, Evaluation, Psychische Erkrankung, Krankenhausbettenkapazität, Verweildauer

## Abstract

The current article addresses, from different perspectives, the question of the optimal number of psychiatric hospital beds required. It summarizes reviews of expert opinions and estimates of optimal bed numbers, considers the balance between inpatient care and other institutions and services for people with mental disorders, addresses associated issues such as the length of stay, and outlines the importance of the local context. Furthermore, it presents the different objectives of bed provision and concludes by presenting a way forward, utilizing data on populations, patients, services, and treatments at a local level.

## Abstract (de)

Der Beitrag betrachtet aus verschiedenen Perspektiven die Frage nach der optimalen Anzahl psychiatrischer Krankenhausbetten. Er fasst Übersichten von Expertenmeinungen und Schätzungen der optimalen Bettenzahl zusammen, beleuchtet das Gleichgewicht zwischen stationärer Versorgung und anderen psychiatrischen Einrichtungen und diskutiert damit verbundene Fragen wie die Bedeutung der Verweildauer und des lokalen Kontexts. Unterschiedliche mögliche Aufgaben und Effekte psychiatrischer Betten werden dargestellt. Abschließend wird eine Perspektive für die Bettenplanung der Zukunft aufgezeigt, die basiert auf der Nutzung von Daten zu Bevölkerung, Patienten, Einrichtungen und Behandlungen auf lokaler Ebene.

The question of how many psychiatric hospital beds are needed has frequently been posed and debated by policy makers, politicians, insurance companies, managers, commissioners, clinicians, and others involved in the planning, funding, and provision of mental health care. Despite an extensive body of literature on this issue since the 1950s, a clear-cut answer to the question—or a formula, preferably based on systematic research evidence—on the precise number of psychiatric beds required has not yet been established, and the debate continues. In this article, I will try to address a few aspects of the question that may inform the debate and help reach an answer, although that answer will not be a number.

## Is there a need for hospital beds?

A fundamental question is whether any psychiatric beds are needed. There have been historical periods and cultures in which psychiatric hospital beds as we know them today did not exist, and these societies did not collapse because of the absence of such beds. Moreover, there are no individually randomized controlled trials showing that psychiatric hospital treatment is more beneficial than no treatment for individuals, nor are there cluster randomized controlled trials demonstrating that geographical areas with psychiatric hospital beds fare better than those without, e.g., whether or not the availability of psychiatric hospital beds is associated with a reduced risk of people with mental disorders harming themselves and others. Thus, there is no research evidence supporting the existence of psychiatric hospital beds.

Despite the absence of evidence, however, hospital beds have been a central component of all psychiatric care since psychiatry was established as a medical discipline at the beginning of the 19th century; today, psychiatric hospital facilities exist in nearly all countries worldwide. One may speculate as to whether, at some point in the future, societies’ response to mental distress in the population may no longer include hospital beds; currently, however, it unequivocally does.

Some trials have shown conventional psychiatric hospital treatment can, to some extent, be replaced by crisis and home treatment teams in the community and by acute day hospitals, but these trials did not include all patients who would typically be hospitalized. Their evidence may be used in arguing for fewer beds but not for abandoning them altogether. One may conclude that some beds are needed, at least for the time being.

## Experts arguing for more or fewer beds

Usually, the question is raised based on the assumption that in the given context there are either too many or too few beds, and that the bed capacity should be adjusted accordingly.

In a systematic review, Mundt et al. [1] summarized the main reasons put forward by experts arguing for more or for fewer beds. Experts in favor of maintaining or increasing the current bed numbers point to a high demand for beds with high occupancy rates, increasing admission numbers, and inappropriately short lengths of stay; the potential criminalization of patients who should be hospitalized but are not; and the lack of alternative care in the community. Central to the argument for more beds is the notion that the demand for beds and their use at full capacity reflects a genuine need for them. This notion has been challenged by the suggestion that, in health care, supply creates demand and there is a tendency for existing beds to always be filled as long as costs are covered.

Experts in favor of reducing the number of beds argue that inpatient services are used for patients who do not need to be there, and that an expansion or better use of community services as well as a better integration of in- and outpatient services would make hospital beds redundant. The implicit or explicit assumption behind this perspective is that care outside hospitals is more beneficial or less costly or both. In the context of modern and well-financed mental health care, this assumption can also be challenged. The conditions in many hospitals today are more accommodating, pleasant, and acceptable than those in the asylums of the 20th century. They intend to be therapeutic and not merely custodial settings. They may well have a beneficial effect similar to care at home. Moreover, the costs are not automatically defined by the setting. For both in- and outpatient treatment, the costs depend on how the treatment program is designed and how resource-intensive its provision is.

## Estimates for needed beds

Using the Delphi method, Mundt et al. sought to find a consensus among 65 experts from six world regions and different backgrounds on the optimal and minimum number of beds [2]. The numbers for a population of 100,000 were 60 beds for an optimal provision and 30 as a minimum. The optimal number is similar to the average number of beds in countries within the Organisation for Economic Co-operation and Development, which is 62 [3]. However, given the wide variation across countries, the number is much higher than what is provided in most low- and middle-income countries and in some high-income countries such as the United Kingdom and Italy, and it is much lower than what is common in other high-income countries such as Japan and Germany.

In a further systematic review and meta-analysis, Mundt et al. [4] identified estimates for the number of beds needed, analyzed their changes over time, and compared these estimates with the actual numbers of beds provided in the corresponding country or region. The reviewed estimates were based on different approaches. They included expert opinions, the use of available data on psychiatric morbidity and service provision in a given area, and the transfer of experiences across different areas with similar characteristics. Historically, the estimates decreased until about 2000 and remained consistent after that. For the period since 2000, the median estimates for beds per 100,000 population were 47 for beds with unspecified length of stay, 28 for short-stay beds, and 10 for long-stay beds. Estimates were about 1.8 times higher than the actual bed numbers in the same area. The authors identified a tendency for an increasing gap between estimates and actual bed numbers. This tendency may indicate that in many regions there is an increasing shortage of psychiatric beds or that the estimates have become more unrealistic over time and fail to reflect changes in actual service provision, including a shift toward alternatives to inpatient care or both.

## The role of other institutions

In 1939, Penrose published a hypothesis that there is a balance between the size of the prison population and the number of psychiatric hospital beds. The larger the prison population, the lower the number of beds and vice versa. Various studies have addressed this hypothesis with inconclusive findings. Some studies suggest a negative association between prison populations and bed numbers, others do not [for literature, see 5].

More generally, a number of studies have explored whether the reductions in psychiatric bed numbers since 1990 have been associated with an increase in other forms of institutionalized care for people with mental disorders, not only prisons, but also forensic services and different forms of supported housing with restricted autonomy for patients (e.g., [5]). In many countries, the prison population and the number of places in forensic services and supported housing schemes have substantially increased since 1990. One question is whether the reduction in beds during de-institutionalization was compensated by an increase in other forms of institutionalized care, reflecting trans-institutionalization, or whether there has been re-institutionalization with an overall increase in the provision of institutionalized care. Data on this question have been collected and analyzed for different world regions. In general, there is a tendency toward both a decrease in psychiatric hospital beds and an increase in other institutions that host people with mental disorders either exclusively, such as forensic services and supported housing schemes, or partly, such as prisons. Whether there is a direct connection between the two processes, however, and, if so, what the exact reasons for the connection are, remains unclear.

This issue points to a wider problem in the debate about which factors influence bed numbers. Ideally the consideration of different factors should be based on empirical evidence. Such evidence, however, requires the analysis of time series of reliable, detailed, and extensive historical data. Such analyses are complex, and the necessary data are rarely available. As a consequence, there are only very few studies analyzing proper time series in different countries, e.g., indicating the influence of economic factors on changes in bed numbers [6]. My personal experience with extensive research on processes of institutionalized care has been that even today it is very difficult, if not impossible, to obtain reliable data on the number of different types of hospital beds and forensic services, and even more so of supported housing schemes or of people with mental disorders in prisons. Even when some of these data can be obtained, the types and definitions of beds and other services vary substantially across countries, making comparisons difficult.

## The role of care in the community

Assuming that there is a certain number of people with mental disorders in a given catchment area, then there should be a balance between inpatient and outpatient care. The more patients are treated in the community, the fewer inpatients should require hospital treatment, either because exacerbations of disorders are prevented through ongoing outpatient care or because acute crises can be dealt with through crisis and home treatment teams instead of leading to a hospital admission—or so the theory goes. In reality, the situation is more complex. Crisis teams can increase the demand for hospital admissions when they come into contact with people identified as needing acute inpatient care who would not have been identified and admitted without the involvement of the crisis service. And the rule in health care that supply creates demand applies not only to hospital beds but also to community services, so that increasing the provision of community services is likely to increase the number of patients served rather than only preventing hospital admissions among those who would otherwise have been admitted. Moreover, larger community services with more capacity can change and shift their focus away from patients with severe illnesses, so that they provide care for those with more common mental disorders rather than prevent hospital admissions in severely ill patients.

A specific characteristic of psychiatric inpatient care is that—unlike other medical specialties—it provides care also on an involuntary basis. Patients are involuntarily admitted, and neither they nor the hospital can avoid the use of hospital beds in these cases. It has been argued that these involuntary admissions and treatments can be reduced by providing involuntary care in the community. While research on many interventions in mental health care is insufficient and the evidence base is ambiguous at best, in this case there has been methodologically rigid research. Randomized controlled trials testing whether involuntary outpatient treatment reduces hospitalizations as compared to voluntary treatment are ethically, legally, and practically difficult to conduct, but such trials have been performed. The results are clear: Hospitalization rates are practically identical between patients in experimental and control groups, and, as far as they have been assessed, other clinical and social outcomes do not show significant differences either. Whatever the reasons for this absence of an effect, the current evidence suggests that involuntary outpatient treatment does not reduce hospitalizations [7].

## Associated questions

The number of beds is usually taken as an indicator of the capacity of a system to treat patients. However, it is not the only indicator. The number of hospital admissions indeed depends on the number of beds, but equally on the average length of stay. The two parameters are directly linked. If the average length of stay is reduced by 10%, the capacity to admit patients is increased by about 11%, and if the length of stay is halved, the capacity is doubled. The question of the length of stay is less often debated and less extensively addressed in the literature than the issue of bed numbers, but it is of similar relevance. Lengths of stay have become shorter over the past few decades in most countries, although they still vary considerably. Normally, services consider briefer stays preferable and aim to shorten them. Two factors may have to be considered in this context. One is that very short stays of between 1 day and a few days lower the average length of stay but often reflect a failure of engagement and therefore a waste of time and resources for both the patients and the health-care system. The other is that the average length of stay can be dominated by patients who remain in hospital a very long time only because no appropriate housing can be found for them. In the latter case, the capacity of the hospital to admit and treat patients is heavily influenced by the capacity of the social care system to provide accommodation for patients who cannot be discharged to independent living without further support.

The question of the optimal length of stay touches on a more fundamental one: What is the precise purpose of inpatient care? Does it function primarily as a respite, as a temporary separation of the patient from a potentially too stressful living situation outside the hospital, and as a way to reduce the burden of caring families for a while? Or is the hospital a setting for active treatment? Although it may be both, the prevailing assumption in health policy and clinical approaches is that active treatment should be provided. If this is so, and if treatment is not exclusively the administration of medication, then one may wonder why in most hospitals treatments are paused on 2 days per week. On weekends, there are often no psychological treatment sessions, whether one-to-one or in groups, and no arts or occupational therapies. More intensive and continuous treatment in hospitals may or may not lead to shortened lengths of stay, but if it does, it will also increase capacity.

## The local context

So far, the article has addressed bed numbers in general, as if the same number of beds would be appropriate or required across different localities. However, local contexts vary in a number of characteristics that are relevant to the discussion about bed numbers. Most importantly, social determinants of mental disorders show substantial variations. A higher psychiatric morbidity should be a reason for more psychiatric hospital beds, and the psychiatric morbidity is driven by social determinants [8]. Mental health disorders are driven by income and wealth inequality; poverty; adverse upbringing conditions; persecution, war, and torture; and social isolation. Incidence rates of psychosis specifically are much higher in deprived urban areas than in affluent rural ones. And in a study conducted in the United Kingdom, being in London compared to other parts of the country was identified as a main factor associated with clinicians initiating involuntary hospital admission [9]. The reasons for these differences are not fully understood, but they indicate that social determinants of mental disorders influence the level of psychiatric morbidity. Social determinants vary substantially not only between world regions and countries but also within countries and even within cities or smaller regions. Thus, considering the local context is essential in a debate about the need for psychiatric beds.

## Needed—for what?

The term “needs” is widely used in health-care planning. It is an ambiguous term that requires further specification to be useful. The first specification concerns the purpose—i.e., the aim—for which something is needed. The second concerns whether what is provided actually achieves that aim. If beds—or other interventions—do not achieve their intended purpose, there is no need for them. Nobody needs ineffective interventions. In this article, only the first specification and the aim are addressed. What are beds for? This question is particularly relevant in the context of well-funded health-care systems, in which hospitals are not always but often well-built and well-equipped places with spacious and clean facilities, friendly staff, and comprehensive care. Unlike during the era of asylums, one can hardly argue that these places are inhumane, and for most patients, staying in them is unlikely to be harmful. Thus, if a society can afford many beds, what is a reason for not providing them? There are at least three potential reasons:

- The most important reason is costs. The provision of inpatient care is expensive, and alternative care can often be provided at lower costs. Thus, there is either a chance to save money or—if mental health-care expenditure remains consistent—to invest in other services that provide more efficient treatment for a larger number of patients.
- A second potential reason is patient preference. Alternative acute treatments to inpatient care can be home treatment or acute day hospitals (historically there were also night hospitals but they seem to have disappeared). Ideally, patients could choose among them and decide which they regard as most acceptable and helpful in their given situation. The availability of such a choice may also reduce involuntary hospital admissions, as some patient who would not want to be treated in a hospital may accept home treatment or a day hospital, depending on how these services are provided. I am not aware of regions where acutely ill patients are presented with such a choice of three different treatment settings.
- A further reason is the current labor market, which keeps changing. In many European countries, there is a shortage of mental health professionals. The specific groups of professionals in short supply vary across countries, but overall, nurses appear to be particularly scarce. There are not enough nurses either generally available on the labor market or specifically willing to work in hospitals with shifts and a limited range of tasks. Working in community teams can be perceived as more variable, more interesting, and more rewarding. When there are not enough nurses to provide high-quality inpatient care, then reducing bed numbers is a way to achieve the desired staff–patient ratio in hospitals.

## Values and perspectives

What then is the general aim of providing psychiatric hospital beds? There are different perspectives linked to different values:

- In a societal and political climate that fosters the commodification of health, beds can be seen as a lucrative setting to generate income and profits for provider organizations. Following this perspective, the number of beds required depends on what health insurance companies and public commissioners are willing to fund and what can be operated with sufficient profit margins. The more the better, and as long as there is no evidence that more beds are harmful, the logic of profit maximizing prevails. The concern that this may be a waste of precious money, assuming that expenditure on health care is limited, and that the money is missing elsewhere in health care would be irrelevant. If the aim is to generate revenue, one could argue that there simply is no predefined limit to health-care expenditure, and that growth of a sector generating income and also providing employment must be a good thing.
- A different aim is to reduce the economic costs of mental disorders for society as a whole. This requires a detailed analysis of loss of productivity for patients, relatives, and others involved in patients’ care, as well as the costs of all health and social care components. Having such a picture based on—hitherto not available—comprehensive data would then allow one to consider the costs and effects of hospital care and decide which bed number represents the optimal efficiency for psychiatric inpatient care.
- From another perspective, one may recognize that a precise number of needed beds does not exist. The aim can be to find a balance between minimizing the expenditure on beds but simultaneously satisfying the ideas and perceptions of other stakeholders in the political debate. This means there should be enough beds to avoid scandals that may be attributed to insufficient bed numbers, and as many—but not more—as the most important political players deem necessary. Since different players can have different ideas about this, it is an ongoing balancing act of political pragmatism.
- Finally, one may aim to provide “optimal” care to reduce the distress of people with mental disorders, of their relatives, and of the local community as much as possible. From this perspective, more expenditure on health care is always welcome, but in a given situation an upper limit is accepted, and plans are made on how to spend the available resources most efficiently. This is arguably the dominant perspective in the professional debate.

Each of these aims and perspectives—and there are potentially more—are legitimate, although one or more of them may be rejected on moral grounds. In debates, they are commonly kept rather implicit, vague, or even mixed. Clarification and awareness of what one would like to achieve with hospital beds may facilitate a fruitful debate about the numbers needed to meet these goals.

## A way forward

Psychiatric hospital beds exist, and decisions about their numbers need to be made daily. How can this be done in a rational way when no undisputed number of needed beds exists, when there are different perspectives and aims, and when the local context as a major factor varies and can have unique features?

This can hardly be discussed by focusing solely on the hospital and ignoring the local context, including all patients with mental disorders in the local health-care sector as well as other services in the region. One potential way forward is to collect, analyze, and present data at the regional level so that they can inform a meaningful debate among all relevant local stakeholders. Ideally, such data should include:

- Demographic characteristics of the local population. This should include data on marginalized and vulnerable groups such as homeless people and refugees.
- Personal characteristics of all patients with mental disorders in the health and social care system.
- Pathways of patients through the system. This is essential to understand the effects of service developments and changes. As an example: When a psychiatric intensive care unit is closed, patients with severe psychosis may be transferred to normal wards; as a result, patients with less severe psychosis are moved to a day hospital; and patients with depression previously treated in the day hospital are discharged. If some of these patients subsequently die by suicide in the community, a connection between suicides of outpatients with depression and the closure of the intensive care unit becomes transparent only when the whole system is considered and pathways are detailed.
- The premises of existing services in the region with the number and qualification of staff.
- The treatments provided, including pharmacological, psychological, and social interventions.
- Outcomes of patients in terms of changes in the severity of illness and their quality of life. The last three aspects are sometimes referred to as quality of structure, process, and outcomes.

Most of these data should be collected routinely and standardized sufficiently across sectors, institutions, and services to be merged and analyzed. Only the data on outcomes require additional activities, which, however, can also benefit the delivery of services and individual treatments. Services worldwide are working to develop outcome assessment systems that provide transparency and facilitate evaluation, the results of which can inform debates about which local services are needed. A proposal for assessing such outcomes in a relatively simple manner has recently been published for the German health-care system [10].

Once all these data are available, a body or organization is still needed to consider the data, discusses them in light of their own values and local circumstances, and reach and authorize decisions about how many beds should be provided in the given region. Establishing such bodies is a political issue, much easier to implement in state-run national health services such as in Italy or the United Kingdom than in more fragmented semi-private systems such as in Germany or Switzerland. Yet, even for the latter countries, it may be the best way forward.

## Practical conclusion

- Whilst there are multiple perspectives and views on the question of the optimal number of psychiatric hospital beds, there is no evidence supporting a single definitive answer.
- For considering the optimal number of beds, the availability of other psychiatric institutions and community-based services is a relevant factor, but the interplay between the function of hospital beds and other services is complex, and the provision of beds is driven by various factors other than the availability of alternative services.
- Bed availability depends on the length of inpatient stays and is strongly influenced by the local context, in particular social factors.
- A way forward can be based on obtaining and utilising detailed data on populations, patients, services, and treatments at a local level to inform service planning and decision-making.

## Declarations

For this article no studies with human participants or animals were performed by any of the authors. All studies mentioned were in accordance with the ethical standards indicated in each case.
